# Green Synthesis of Metallic Nanoparticles via Biological Entities

**DOI:** 10.3390/ma8115377

**Published:** 2015-10-29

**Authors:** Monaliben Shah, Derek Fawcett, Shashi Sharma, Suraj Kumar Tripathy, Gérrard Eddy Jai Poinern

**Affiliations:** 1 Murdoch Applied Nanotechnology Research Group, Faculty of Minerals and Energy, School of Engineering and Energy, Murdoch University, Murdoch WA 6150, Australia; monalinikunj.shah@gmail.com (M.S.); D.Fawcett@murdoch.edu.au (D.F.); 2 Biosecurity and Food Security Academy, School of Veterinary and Life Sciences, Agricultural Sciences Murdoch University, Murdoch WA 6150, Australia; S. Sharma@murdoch.edu.au; 3 School of Biotechnology, School of Applied Sciences, KIIT University, Campus-11, Bhubaneswar 751024, Odisha, India; suraj.tripathy@kiitbiotech.ac.in

**Keywords:** green chemistry, biological synthesis, nanoparticles

## Abstract

Nanotechnology is the creation, manipulation and use of materials at the nanometre size scale (1 to 100 nm). At this size scale there are significant differences in many material properties that are normally not seen in the same materials at larger scales. Although nanoscale materials can be produced using a variety of traditional physical and chemical processes, it is now possible to biologically synthesize materials via environment-friendly green chemistry based techniques. In recent years, the convergence between nanotechnology and biology has created the new field of nanobiotechnology that incorporates the use of biological entities such as actinomycetes algae, bacteria, fungi, viruses, yeasts, and plants in a number of biochemical and biophysical processes. The biological synthesis via nanobiotechnology processes have a significant potential to boost nanoparticles production without the use of harsh, toxic, and expensive chemicals commonly used in conventional physical and chemical processes. The aim of this review is to provide an overview of recent trends in synthesizing nanoparticles via biological entities and their potential applications.

## 1. Introduction

In recent years, the convergence of nanometre size scale technologies and biological technologies has created the new field of nanobiotechnology. This relatively new field is focused on the creation, manipulation, and use of materials at the nanometre scale for advanced biotechnology [1]. At the forefront of this field is the synthesis of nanometre size scale particles via biological entities. Nanoparticles are of great interest due to their novel physicochemical, magnetic, and optoelectronic properties that are governed by their size, shape, and size distribution [2,3,4,5,6]. It is predominantly the nanoparticles’ extremely small size and large surface area to volume ratio that leads to the significant differences in properties (e.g., biological, catalytic activity, mechanical properties, melting point optical absorption, thermal and electrical conductivity) not seen in the same material at larger scales in their bulk form [7]. Because of these unique physicochemical and optoelectronic properties, nanoparticles are of particular interest for a number of applications ranging from as catalysts, chemical sensors, electronic components, medical diagnostic imaging, pharmaceutical products, and medical treatment protocols. For example, metallic nanoparticles of noble metals such as gold, silver, platinum, and palladium have been widely used in products ranging from cosmetic to medical and pharmaceuticals. Gold nanoparticles have been extensively used in biomedical applications [8,9,10], separation sciences [11], disease diagnostics [12], and pharmaceuticals [13,14]. Silver nanoparticles have been found to possess both anti-bacterial and anti-inflammatory properties that can promote faster wound healing. Because of these advantageous properties, silver nanoparticles have been integrated into commercially available wound dressings, pharmaceutical preparations, and medical implant coatings [15,16,17,18,19,20]. Platinum nanoparticles have been widely used in biomedical applications in either pure form or alloyed with other nanoparticles [21] and palladium nanoparticles in catalysis and electro-catalysis applications [22,23,24], chemical sensors [25], optoelectronics [26], and anti-bacterial applications [27]. In addition, non-noble metallic nanoparticles such as iron [28,29], copper [30], zinc oxide [31], and selenium [32] have also been used in medical treatments, cosmetic formulations, and anti-bacterial applications.

Due to the increased demand for various metallic and non-metallic nanoparticles over the past two decades, a wide range of physical and chemical techniques have been developed to produce nanoparticles of different sizes, shapes, and compositions. Traditionally, nanoparticles have been synthesized and stabilised via physical and chemical techniques. The physical approach includes techniques such as laser ablation [33], lithography [34] and high-energy irradiation [35]. While the chemical approach uses techniques such as: chemical reduction, electrochemistry, and photochemical reduction [36,37,38,39,40]. Studies have shown that during the synthesis process, size, shape, stability, and physicochemical properties of the nanoparticles are strongly influenced by a variety of factors. These factors include process parameters (temperature, concentrations, *etc.*), process kinetics involving the interplay between the metal ion precursors and the reducing agent, and adsorption kinetics involving the stabilizing agent and the nanoparticles [41,42]. Consequently, designing a process that effectively controls the size, shape, stability, and physicochemical properties is currently at the forefront of research into nanoparticle synthesis [43,44]. Conventional synthesis of nanoparticles can involve expensive chemical and physical processes that often use toxic materials with potential hazards such as environmental toxicity, cytotoxicity, and carcinogenicity [45]. The toxicity problems arise from the hazardous substances, such as organic solvents, reducing agents, and stabilizers that are used to prevent unwanted agglomeration of the colloids. In addition, some nanoparticles have also been found to be toxic due to factors such as composition, size, shape, and surface chemistry. As a result, the presence of these toxic formation agents on the synthesized nanoparticles and potentially the nanoparticles themselves has prevented their clinical and biomedical application. Importantly, all these factors can be potentially controlled via biological mediated production. As a result, there is currently widespread interest in developing clean, reliable, biologically compatible, benign, and environment-friendly green processes to synthesize nanoparticles [44,46].

In recent years, biological synthesis has emerged as an attractive alternative to traditional synthesis methods for producing nanoparticles. Biosynthesis involves using an environment-friendly green chemistry based approach that employs unicellular and multicellular biological entities such as actinomycetes [47,48], bacteria [49,50,51,52,53], fungus [54,55,56,57], plants [58,59], viruses [60,61], and yeast [62,63,64]. Synthesising nanoparticles via biological entities acting as biological factories offers a clean, nontoxic and environment-friendly method of synthesizing nanoparticles with a wide range of sizes, shapes, compositions, and physicochemical properties [65]. Another interesting feature of many biological entities is their ability to act as templates in the synthesis, assembly and organisation of nanometre scale materials to fabricate well-defined micro and macro scale structures. For example, viruses have been used to assemble gold and iron oxide nanoparticles to form microstructures [66], bacteriophages have also been used to form intricate nanometre and micrometre scale structures [67,68,69] and phage based assemblies of liposomes have been used in targeted drug delivery procedures [70,71,72,73]. Comparing the above-mentioned biological identities and their potential to become efficient biological factories, synthesizing nanoparticles via plants, is a relatively straight forward and advantageous approach [74,75]. In comparison with microorganisms, the plant approach is more advantageous since it does not need any special, complex, and multi-step procedures such as isolation, culture preparation, and culture maintenance. Furthermore, synthesis in plants tends to be faster than microorganisms, is more cost-effective and is relatively easy to scale up for the production of large quantities of nanoparticles [74,76,77,78,79]. The aim of this review is to present a brief overview of the techniques used to characterise nanoparticles, microbial routes for synthesising metal and metal oxide nanoparticles, use of plants extracts for synthesis of nanoparticles, factors influencing the synthesis process, possible mechanisms involved in nanoparticle formation and growth, and potential applications of nanoparticles synthesised using natural biological factories found in plants.

## 2. Characterisation Techniques

To date, there are numerous techniques for synthesizing nanoparticles. However, these techniques fall into two broad approaches and can be defined as either a top down approach or a bottom up approach [80,81,82]. The top down approach starts with a material of interest, which then undergoes size reduction via physical and chemical processes to produce nanoparticles. Importantly, nanoparticles are highly dependent on their size, shape, and surface structure and processing tends to introduce surface imperfections. These surface imperfections can significantly impact on the overall nanoparticle surface physicochemical properties [83]. In the bottom up approach, nanoparticles are built from atoms, molecules and smaller particles/monomers [84,85,86]. In either approach, the resulting nanoparticles are characterized using various techniques to determine properties such as particle size, size distribution, shape, and surface area. This is of particular importance if the properties of nanoparticles need to be homogeneous for a particular application.

In the case of chemical and biological synthesis of nanoparticles, the aqueous metal ion precursors from metal salts are reduced and as a result a colour change occurs in the reaction mixture. This is the first qualitative indication that nanoparticles are being formed. One interesting property of colloidal particles in solution, due to their size and shape, is their ability to be seen when a laser beam passes through the colloidal solution. This effect is known as the Tyndall effect and is a simple and straightforward technique that can be used to detect the presence of nanoparticles in solution [87]. After the reaction, nanoparticles can be separated from the colloid by high speed centrifugation and then examined using advanced nanocharacterization techniques.

Some of the spectroscopy and microscopy techniques routinely used include UV-visible spectroscopy (UV-vis), dynamic light scattering (DLS), atomic force microscopy (AFM), transmission electron microscopy (TEM), scanning electron microscopy (SEM), energy dispersive spectroscopy (EDS), powder X-ray diffraction (XRD), Fourier transform infrared spectroscopy (FT-IR), and Raman spectroscopy. Microscopy based techniques such as AFM, SEM and TEM are considered direct methods of obtaining data from images taken of the nanoparticles. In particular, both SEM and TEM have been extensively used to determine size and morphological features of nanoparticles [87,88,89,90].

Spectroscopy based techniques such as UV-vis, DLS, XRD, EDS, FT-IR, and Raman are considered indirect methods of determining data related to composition, structure, crystal phase, and properties of nanoparticles. The UV-visible spectroscopy covers the UV range between 190 and 380 nm and the visible range between 380 and 800 nm. Both types of radiation interact with matter and promote electronic transitions from the ground state to higher energy states. Wavelengths between 300 and 800 nm are generally used for characterizing metallic nanoparticles ranging in size from 2 nm up to around 100 nm [87]. For example, absorption measurements for silver (Ag) nanoparticles are usually between 400 and 450 nm [91,92], while gold (Au) nanoparticles are generally detected by the presence of peaks between 500 and 550 nm [93,94]. DLS spectroscopy can be used to determine size distribution and quantify the surface charge of nanoparticles suspended in a liquid [87,95]. The elemental composition of nanoparticles can be determined via EDS mapping. Whereas XRD examination produces a diffraction pattern that is subsequently compared with data contained in a standard crystallographic database to determine structural information. Analysis of the XRD data identifies crystallite size, structure, preferred crystal orientation, and phases present in samples [96,97]. FT-IR spectroscopy can be used to investigate surface chemistry and identify surface residues such as functional groups like carbonyls and hydroxyls moieties that attach to the surface during nanoparticle synthesis. Raman spectroscopy is useful in detecting vibrational modes of molecules and can be used to identify vibrational signals of a variety of chemical species that are attached to the surface of nanoparticles during synthesis [87]. For example, using surface-enhanced Raman scattering (SERS) it was possible to measure single molecular attachments on Ag nanoparticles [98].

## 3. Biological Synthesis of Nanoparticles

Recent studies have shown that green biologically based methods using microorganisms and plants to synthesize nanoparticles are safe, inexpensive, and an environment-friendly alternative [99,100]. Both microorganisms and plants have long demonstrated the ability to absorb and accumulate inorganic metallic ions from their surrounding environment. These attractive properties make many biological entities efficient biological factories capable of significantly reducing environmental pollution and reclaiming metals from industrial waste. Importantly, the ability of a biological entity to use its inherent biochemical processes to transform inorganic metallic ions into metal nanoparticles has led to a relatively new and largely unexplored field of research [101]. To date, the ability of microorganisms to interact, extract, and accumulate metallic materials from their surroundings has been capitalized on in a number of biotechnology applications such as bioremediation and bioleaching [102,103]. The capability of microorganisms to actively interact with their surrounding environment stems from the composition of their lipid-based amphipathic membranes enables a variety of oxidation-reduction mechanisms to take place and promote biochemical conversions [104,105,106]. Studies have shown that both unicellular and multicellular organisms achieve both extracellular and intracellular synthesis of inorganic micron and nano-sized materials as presented in Table 1, and in the case of nanoparticle synthesis, culturing microorganisms in particular environments can also assist them in promoting coupled oxidation and reduction phenomenon [104,107]. The specific oxidation-reduction mechanisms, nucleation, and subsequent nanoparticle growth kinetics and the interaction of these processes with the microorganism metabolic processes have yet to be fully explained [108,109,110,111]. Hence, there is still a considerable level of research that needs to be undertaken to fully investigate and elucidate differences in nanoparticle size and morphology between different metals when synthesized using the same microorganism [65,105]. This is also true when considering the use of plants for synthesizing nanoparticles. The advantage of using plants over other eco-friendly biologically based systems such bacteria and fungi, is that it avoids the use of specific, well-conditioned culture preparation and isolation techniques that tend to be expensive and elaborate. Conversely, biosynthesis of nanoparticles using plants or plant based extracts tends to be safe, have relatively short production times, and have a lower cultivation cost compared to other biological systems [112]. Furthermore, plant based biosynthesis is a relatively straightforward process that can be easily scaled up for large-scale production of nanoparticles.

As mentioned above, nanoparticles can be synthesised from a wide variety of biological entities such as actinomycetes, algae, bacteria, fungus, plants, viruses, and yeast. Each biological entity has varying degrees of biochemical processing capabilities that can be effectively used to synthesize particular metallic or metallic oxide nanoparticles. Not all biological entities can synthesize nanoparticles due to their enzyme activities and intrinsic metabolic processes. Therefore, careful selection of the appropriate biological entity is necessary to produce nanoparticles with well-defined properties such as size and morphology. Generally, biological entities with a potential to accumulate heavy metals have the best chance of synthesizing metallic nanoparticles [113,114,115,116]. In the case of a microorganism, culturing methods are very important. Hence optimisation of culturing parameters such as nutrients, light, medium pH, temperature, mixing speed, and buffer strength can significantly increase enzyme activity [74,117]. Recently, the biological synthesis of nanoparticles using plants and plant extracts appears be to an attractive alternative to conventional chemical synthesis and the more complex culturing and isolation techniques needed for many microorganisms. Moreover, combinations of molecules found in plant extracts perform as both reducing and stabilizing (capping) agents during nanoparticle synthesis [118,119,120]. These biological molecules are chemically complex, but have the advantage of being environment-friendly.

**Table 1:** A selection of microorganisms used to synthesize nanoparticles.

Microorganism	Nano Particle	Size (nm)	Extracellular/Intracellular	Reference
Actinomycetes	-	-	-	-
*Rhodococcus* sp.	Au	5 to 15, Spherical	I	[93]
*Thermomonospora* sp.	Au	8, Spherical	E	[47,48]
Algae	-	-	-	-
*Chlorella vulgaris*	Au	40 to 60, Spheroid, polyhedral	I	[121]
*Sargassum wightii*	Au, Ag	Spheroid	E	[122]
Bacteria	-	-	-	-
*Escherichia coli*	CdS	2 to 5, Spherical	I	[123]
*Pseudomonas aeruginosa*	Au	15 to 30 Spherical	E	[53]
*Pseudomonas stutzeri*	Ag	Up to 200, various shapes	I	[109]
Fungus	-	-	-	-
*Aspergillus flavus*	Ag	8 to 10 Spherical	I	[124]
*Colletotrichum* sp.	Au	20 to 40 Spherical	E	[125]
*Fusarium oxysporum*	Au	20 to 40, Spherical, triangular	E	[126]
*Volvariella volvacea*	Ag & Au	20 to 150, Spherical, hexagonal	E	[127]
Viral	-	-	-	-
*M13 bacteriophage*	CdS, ZnS	Quantum dots, nanowires	E	[128]
*M13 bacteriophage*	HAP	Hydroxyapatite fibrils	E	[129,130]
Bacteriophage	Ca	Fibrils		[131,132]
Tobacco mosaic virus (TMV)	Silica	Various shapes	E	[133,134]
Tobacco mosaic virus (TMV)	SiO_2_, CdS, PbS, Fe_2_O_3_	Nanotubes on surface	E	[60,135]
Yeast	-	-	-	-
*Candida glabrata*	CdS	2, Spherical	I	[62]
*Saccharomycetes. cerevisiae*	Sb_2_O_3_	3 to 10, Spherical	I	[77]
*Candida glabrata* (Yeast)	CdS	3 to 100	I	[136]
Yeast strain MKY3	Ag	2 to 5, Hexagonal	E	[63]
Schizosaccharomyces pombe	CdS	1 to 2, Hexagonal	I, I	[62,110]
*Torulopsis* sp.	PbS	2 to 5, Spherical	I	[137]

The importance of developing environment-friendly sustainable metal nanoparticle producing technologies using the principles of green chemistry is discussed. The first part of this review briefly surveys the use of microorganisms and the second, more extensive part, examines the role of plants in synthesizing metal nanoparticles.

## 4. Microbial Routes for Nanoparticle Synthesis

Many studies have shown that microorganisms, both unicellular and multicellular have the ability to synthesize inorganic materials. The biological synthesis can be considered a bottom-up approach where nanoparticle formation occurs due to the reduction/oxidation of metallic ions via biomolecules such as enzymes, sugars, and proteins secreted by the microorganism [138]. However, a complete understanding of nanoparticle synthesis mechanism occurring in microorganisms is yet to be fully developed. This is because each type of microorganism tends to behave and interact differently with particular metallic ions. The interaction and biochemical processing activities of a specific microorganism and the influence of environmental factors such as pH and temperature ultimately determines the formation of nanoparticles with a particular size and morphology [50,100]. Nanoparticle formation can be either extracellular or intracellular depending on the microorganism as seen in Table 1 [139,140,141,142,143]. The following six sections briefly discuss some of the main microbial routes used to synthesise nanoparticles.

### 4.1. Actinomycetes

The literature reports extensively on the extracellular or intracellular synthesis of metallic nanoparticles via actinomycetes [144,145,146], with extracellular synthesis being the more common pathway. Intracellular reduction of metallic Au ions by the *Rhodococcus* sp. has revealed that Au nanoparticles were predominantly reduced on the cell membrane and cell wall, but not in the cytosol. Reduction of Au ions is believed to be the result of interacting enzymes being released from the cell membrane and cell wall while capping proteins stabilizes the formed nanoparticles. The biosynthesis process produced mono-dispersed Au nanoparticles ranging from 5 to 15 nm in size; the nanoparticles were non-toxic to the cell [144]. Similar studies with actinomycete cells have confirmed the intracellular reduction of Au and Ag ions by cell wall enzymes to form metallic Ag seeds/monomers that consequently initiate the growth of nanoparticles [147,148,149,150].

In an effort to explain the mechanism and conditions that favoured extracellular synthesis of nanoparticles in 2014, Karthik *et al.* undertook the reduction of silver nitrate (AgNO_3_) ions by using *Streptomyces* sp. LK-3. This resulted in the efficient formation of Ag nanoparticles [145]. It is known that the nitrate reductase enzyme is generally involved in the cellular nitrogen cycle and is responsible for the reduction of nitrate to nitrite [151]. Their study indicated that Nicotinamide adenine dinucleotide (NADH-) dependent nitrate reductase enzyme, was indeed responsible for the reduction of Ag ions to metallic Ag via an electron transfer mechanism, and the subsequent formation of stabilized Ag nanoparticles. A similar nitrate reductase enzyme mechanism is seen in the reduction of Au ions from aqueous solutions containing gold chloride (AuCl_4_^−^) ions [152]. During the electron transfer from NADH by NADH-dependent reductase, each Au ion receives an electron and it reduces to Au^0^ and subsequently forms stabilized Au nanoparticles [153,154]. Importantly, effective stabilization is necessary to prevent agglomeration due to the high-surface energy and protect the properties of the synthesized nanoparticle. Interestingly, biologically synthesized nanoparticles tend to have higher antimicrobial activity when compared with traditionally synthesized nanoparticles. The higher antimicrobial activity is believed to be the result of the action of synergistic proteins involved in capping and stabilizing the nanoparticles [155].

### 4.2. Algae

Algae are aquatic microorganisms and recent studies have shown that some of them not only accumulate heavy metals, but they can also be used to biologically synthesize metallic nanoparticles. For example, the dried unicellular alga *Chlorella vulgaris* was used to synthesize tetra-chloroaurate ions to form algal-bound gold that was subsequently reduced to form Au nanoparticles. The tetrahedral, decahedral and icosahedral shaped nanoparticles were found to accumulate near the cell surfaces [121]. A similar study using an extract from *C. vulgaris* was found to produce Ag nanometre scale plates at room temperature. The study indicated that proteins contained within the extract acted as reducing agent, shape-control modifier and stabilizing agent [156]. And a study by Govindaraju *et al.* revealed that a marine alga *Sargassum wightii* was capable of extracellular synthesis of Au, Ag and Au/Ag bimetallic nanoparticles [157]. Recently, Singaravelu *et al.* showed that *S. wightii* could rapidly synthesize Au nanoparticles. The extracellular synthesis produced nanoparticles ranging in size from 8 to 12 nm [122]. Rajasulochana *et al.* have also reported the synthesis of extracellular Au nanoparticles using *Kappaphycus alvarezii* [158]. While Mata *et al.* has reported on the biological reduction of Au using biomass derived from brown alga *Fucus vesiculosus* [159]. Additionally, Senapati *et al.* reported the intracellular synthesis of Au nanoparticles via *Tetraselmis kochinensis* [160]. And recently, Castro *et al.* reported using red *Chondrus crispus* and green alga *Spirogyra insignis* for synthesizing Au and Ag nanoparticles [161].

### 4.3. Bacteria

In nature, bacteria are frequently exposed to diverse and sometimes extreme environmental situations. Survival in these harsh conditions ultimately depends on their ability to resist the effects of environmental stresses. Natural defence mechanisms exist in bacteria to deal with a variety of stresses such as toxicity arising from high concentrations of metallic ions in the environment. Biological strategies for dealing with high concentrations of metallic ions include changes in metal ion concentration via redox state changes, efflux systems, intracellular precipitation, and accumulation of metals, and extracellular formation of complexes [162]. The major bacterial species used for the synthesis of metallic nanoparticles include *Actinobacter* sp., *Escherichia coli*, *Klebsiella pneumonia*, *Lactobacillus* spp., *Bacillus cereus*, *Corynebacterium* sp., and *Pseudomonas* sp. [65,163,164,165]. Bacteria are known to synthesise metallic nanoparticles by either intracellular or extracellular mechanisms. For example, Ag nanoparticles have been synthesized using *Pseudomonas stutzeri* AG259 bacterium via a mechanism involving the NADH-dependent reductase enzyme that donates an electron and oxidises to NAD^+^. The electron transfer results in the biological reduction of Ag ions to Ag nanoparticles [55]. In a similar study, Husseiny *et al.* were able to reduce Au ions using *Pseudomonas aeruginosa* that resulted in the extracellular synthesis of Au nanoparticles [53]. However, some other researchers have also shown the non-involvement of biological enzymes. For example, Liu *et al.* were able to produce Au nanoparticles from dried cells of *Bacillus megaterium* [166]. A similar study by Sneha *et al.* using a *Corynebacterium* sp also revealed that a non-enzymatic reduction mechanism was involved in nanoparticle formation [167]. The reduction of nanoparticles is believed to be the result of a combination of several factors. The first factor is the presence of some organic functional groups at the cell wall that induce reduction, and the second depends on the appropriate environmental parameters such as pH and temperature being present [168]. For example, the dried biomass of *Lactobacillus* sp. A09 and *Bacillus megaterium* D01 can reduce Ag ions via the interaction of functional groups present on the cell wall to produce silver nanoparticles [169].

Size, shape, and composition of a nanoparticle can be significantly influenced by pH and temperature [170]. For example, particle size is an important factor since novel and unique physicochemical properties are more pronounced at smaller sizes. Therefore, there is a need to optimize synthesis parameters during nanoparticle formation to enhance the overall particle properties. In particular, selecting the appropriate culture media for a specific bacteria and the particular metallic salt is important since these two parameters form the basis of nanoparticle synthesis and can influence particle yield [49,51,171]. Studies by He *et al.* using bacterium *Rhodopseudomonas capsulata* have shown that particle size and morphology can be influenced by both metallic salt concentration and medium pH. At pH 6, dilute concentrations of AuCl_4_ tended to produce spherical Au nanoparticles ranging in size from 10 to 20 nm. Upon increasing the salt concentration, this reaction tended to produce Au nanowires at pH 6 [172]. Also, when the pH was changed to 4, dilute salt concentrations tended to produce both spheres and triangular nanometre scale plates [153]. The studies clearly indicated that controlling medium pH directly influenced nanoparticle morphology during formation. Table 1 summarizes the major bacterial species that have been used to synthesize a variety of nanoparticles along with composition, particle size range, and morphology.

### 4.4. Fungi

Biosynthesis of nanoparticles utilising fungi is widespread among many research groups globally and the synthesis occurs at both extracellular and intracellular locations. For example, fungi such as *Aspergillus* sp., *Fusarium* sp., and *Penicillium* sp. have been frequently reported for their biosynthetic ability to create both Ag and Au nanoparticles [124,125,127,173,174]. Moreover, studies have shown that fungi are capable of producing mono dispersed nanoparticles and particle sizes over a wide range of different chemical compositions as seen in Table 1. Fungi possess some additional attributes when compared to their bacterial counterparts for the synthesis of metallic nanoparticles. For instance, fungi secrete large amounts of proteins and enzymes per unit of biomass, which results in larger amounts of nanoparticles being manufactured [175]. Studies have shown that some fungi possess high intracellular metal uptake volumes and the synthesised particles tend to be smaller in size [126,176]. However, the culture conditions can have a significant influence during the biosynthesis of metallic nanoparticles. For example, the biological reduction of Au ions was carried out using *Trichothecium* sp. biomass under stationary conditions synthesized extracellular nanoparticles. In contrast, agitation of the biomass tended to produce intracellular nanoparticles. One possible explanation suggested by this result was that non-agitation promoted the release of enzymes and proteins while agitation prevented their release [56]. Fluorescence spectra studies have indicated that extracellular synthesis of nanoparticles by the fungi results from the action of bioactive reducing agents secreted from the cell wall and it produces protein-stabilized nanoparticles. The study was able to show that the same proteins released by the fungal biomass were present in the solution and also bound to the surfaces of nanoparticles [5,177,178]. Both extracellular and intracellular synthesis of nanoparticles using fungi has been investigated. In the case of intracellular synthesis, extraction procedures in downstream processing suffer from the drawback of low yields. In contrast, extracellular synthesis produces nanoparticles at the cell surface or at the periphery of the cell, which means they can be readily recovered in downstream processing [162,173]. A very notable feature of some fungi is their ability to synthesize nanoparticles of different chemical compositions. For example, studies by Bansal *et al.* have shown that *Fusarium oxysporum* can biosynthesize silica and titania nanoparticles from aqueous solutions of Si_6_^2−^ and TiF_6_^2−^ respectively [179]. Furthermore, the synthesis of nanometre scale materials such as luminescent CdSe quantum dots [180], magnetite [181], zirconia [182], and oxide nanoparticles [183] have been reported in the literature.

### 4.5. Viruses

The use of viruses in the synthesis of nanomaterials is a novel technique that has been able to deliver inorganic materials such as silicon dioxide (SiO_2_), cadmium sulphide (CdS), iron oxide (Fe_2_O_3_), and zinc sulphide (ZnS). Semiconductor materials such as CdS and ZnS are of particular interest to the electronics industry and green chemistry based methods for their synthesis have been extensively investigated. The use of viruses to synthesize quantum dots has been investigated over the last decade [60,62,128]. An attractive feature of viruses is their dense surface covering of capsid proteins that form a highly reactive surface capable of interacting with metallic ions [100]. For example, a typical plant virus such as the Tobacco mosaic virus (TMV) particle can have as many as 2130 capsid protein molecules covering its surface. The array of proteins can act as attachment points for the deposition of materials [184,185,186,187] or can be used to create three-dimensional vessels for pharmaceuticals [188,189,190]. In a recent study, low concentrations of TMV’s were added to Ag or Au salts before adding plant extracts of *Nicotiana benthamiana* (Round-leaved native tobacco) or *Hordeum vulgare* (Barley). The presence of the virus not only decreased the size of the synthesized nanoparticles, but also dramatically increased their numbers compared to the non-virus solutions [191]. The study also revealed that at higher TMV concentrations fewer free nanoparticles were formed and at the same time the TMV acted as a bio-template that underwent metallization to form nanowires. Similar studies have also shown the potential of viruses to be used as a template for the manufacture of nanometre scale structures such as nanowires and nanotubes [61,135].

### 4.6. Yeasts

Yeasts, like many other microorganisms, have the ability to absorb and accumulate significant amounts of toxic metals from their surrounding environment [105,107]. Adaptation to metal toxicity has resulted in yeast cells using a variety of detoxification mechanisms that cause activities such as chelation, bio-precipitation bio-sorption and extracellular sequestration. The variation in particle size, location, and particle properties is due to the different mechanisms used by yeast organisms to form and stabilise the nanoparticles during synthesis [170]. Studies by Dameron *et al.* have shown that when *Candida glabrata* was exposed to cadmium salts the resulting intracellular synthesis produced CdS quantum dots [62]. Similar studies using *Schizosaccharomyces pombe* cells have also been able to find a link between the formation of CdS quantum dots and the growth phase of the yeast [110,192]. Moreover, intracellular synthesis of PbS quantum dots was possible when *Torulopsis* sp. were exposed to Pb^2+^ ions [137]. Also, Au nanoparticles ranging in size from a few nanometres up to around 100 nm have been intracellularly synthesized using *Pichia jadinii*.

Importantly, it was found that during the synthesis step, the nanoparticle size and shape was easily regulated by controlling both the growth and cellular activities of *P. jadinii* [136]. In similar studies, the influence of biomass and Au salt concentration during extracellular synthesis [193] and intracellular synthesis [194] were studied using the marine yeast *Yarrowia lipolytica*, the studies revealed that both biomass and Au salt influenced the size and morphology of particles formed. For example, increasing Au salt concentration not only continued to produce nanometre scale spheres, but it also tended to produce nanometre scale plates. Furthermore, the silver-tolerant yeast strain MKY3 has been used to synthesize extracellular Ag spherical nanoparticles ranging in size from 2 to 5 nm [63].

## 5. Biological Synthesis of Metal Nanoparticles via Plants

**Figure 1:**
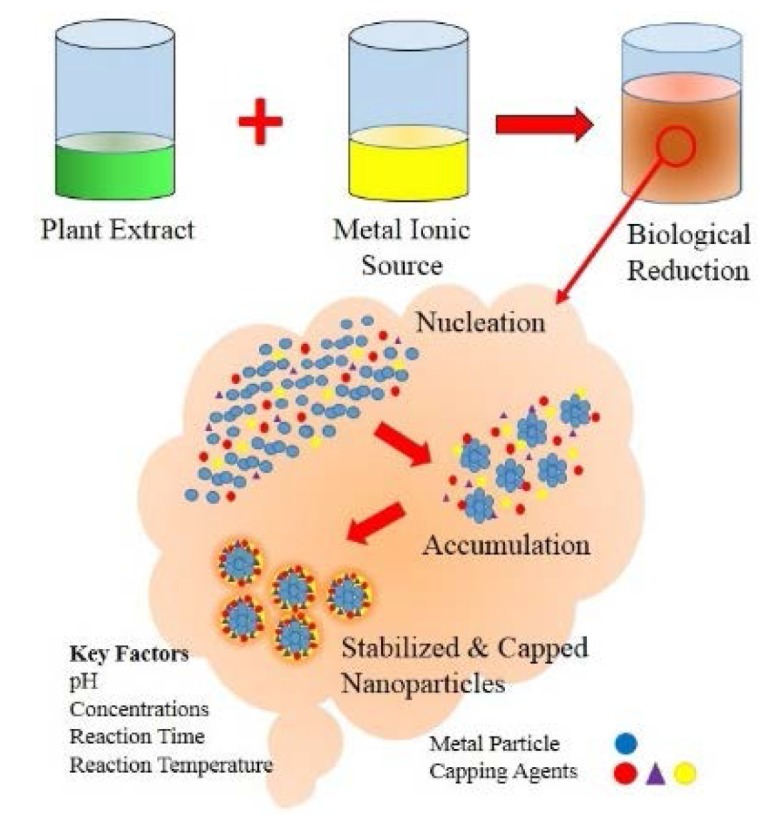
Biological synthesis of nanoparticles using plant extracts.

It has long been known that plants have the potential to hyper-accumulate and biologically reduce metallic ions [44,69]. Because of these interesting properties, plants have been considered a more environment-friendly route for biologically synthesizing metallic nanoparticles and for detoxification applications [66,69]. Plant extracts containing bioactive alkaloids, phenolic acids, polyphenols, proteins, sugars, and terpenoids are believed to have an important role in first reducing the metallic ions and then stabilizing them as seen in Figure 1 [195,196]. The variation in composition and concentration of these active biomolecules between different plants and their subsequent interaction with aqueous metal ions is believed to be the main contributing factors to the diversity of nanoparticle sizes and shapes produced as seen in Table 2 [197]. Importantly, the synthesis of nanoparticles from reducing metal salts via plants is a relatively straightforward room temperature process. The process begins by mixing a sample of plant extract with a metal salt solution. Biochemical reduction of the salts starts immediately and the formation of nanoparticles is indicated by a change in the colour of the reaction mixture. During synthesis, there is an initial activation period when process metal ions are converted from their mono or divalent oxidation states to zero-valent states and nucleation of the reduced metal atoms takes place [198]. This is immediately followed by a period of growth when smaller neighbouring particles amalgamate to form larger nanoparticles that are thermodynamically more stable while further biological reduction of metal ions takes place. As growth progresses nanoparticles aggregate to form a variety of morphologies such as cubes, spheres, triangles, hexagons, pentagons, rods, and wires [23]. In the final stage of synthesis, the plant extracts ability to stabilize the nanoparticle ultimately determines it’s most energetically favourable and stable morphology. Properties of the plants extract such as its concentration, metal salt concentration, reaction time, reaction solution pH, and temperature significantly influence the quality, size, and morphology of the synthesized nanoparticles [112,199].

**Table 2:** A selection of nanoparticles synthesized by various plants.

Plant	Nanoparticle	Size (nm)	Shape	Reference
*Aloe vera*	Au & Ag	50 to 350	Spherical, triangular	[200]
*Aloe vera*	In_2_O_3_	5 to 50	Spherical	[201]
*Camelia sinensis*	Ag, Au	30 to 40	Spherical, triangular, irregular	[202]
*Citrullus colocynthis*	Ag	31	Spherical	[203]
*Curcuma longa*	Pd	10 to 15	Spherical	[120]
*Diopyros kaki*	Pt	15 to 19	Crystalline	[204]
*Eucalyptus macrocarpa*	Au	20 to 100	Spherical, triangular, hexagonal	[84]
	Ag	10 to 100	Spherical, cubes	[92]
*Mangifera indica*	Ag	20	Spherical, triangular, hexagonal	[205]
*Rhododendron dauricum*	Ag	25 to 40	Spherical	[206]
*Psidium guajava*	Au	25 to 30	Spherical	[207]
*Pyrus* sp. (Pear fruit extract)	Au	200 to 500	Triangular, hexagonal	[208]
*Terminalia catappa*	Au	10 to 35	Spherical	[209]

### 5.1. Factors Affecting Biological Synthesis of Metal Nanoparticles

During biological synthesis of metallic nanoparticles, a number of controlling factors are involved in the nucleation and subsequent formation of stablised nanoparticles. These factors include pH, reactant concentrations, reaction time, and temperature. The following sections briefly discuss each of these factors in succession.

#### 5.1.1. Influence of pH

The pH value of the reaction medium plays a significant role during the formation of nanoparticles [210]. Studies have shown that varying the pH of the reaction medium tends to produce variability in shape and size of nanoparticles synthesized. In particular, larger particles tend to be produced at a lower acidic pH values compared to high pH values [211,212]. For example, rod-shaped Au nanoparticles synthesized using *Avena sativa* (Oat) biomass were larger (25 to 85 nm) when formed at pH 2 and relatively smaller (5 to 20 nm) at pH 3 and 4 [213]. The study suggested that between pH 3 and 4 more accessible functional groups contained within the extract were available for particle nucleation. Conversely, at pH 2 fewer functional groups were available and resulted in particle aggregation to form larger Au nanoparticles. In a similar study, Ag nanoparticles were synthesized using *Cinnamon zeylanicum* bark extract and the number of particle synthesized increased with increasing concentrations of bark extract and at higher pH values (pH 5 and above) the shape of the nanoparticles tended to become spherical [214]. On the other hand, when *Cinnamon zeylanicum* bark extract was used to synthesize palladium (Pd) nanoparticles there was a slight increase in particle size with increasing pH. When the pH was less than 5 the particle ranged from 15 to 20 nm and when the pH was greater than 5 particles ranged in size from 20 to 25 nm [215].

#### 5.1.2. Influence of Reactant Concentration

The concentration of biomolecules found in plants extracts can significantly influence the formation of metallic nanoparticles. A study by Huang *et al.* found that by varying the amount of sundried *Cinnamomum camphora* (camphor) leaf extract in the reaction medium could significantly influence the shape of the synthesized Au and Ag nanoparticles [216]. For example, when the precursor chloroauric acid was subjected to increasing concentrations of extract, the resulting nanoparticle shape changed from triangular to spherical. Similarly, varying the amount of *Aloe vera* leaf extract in the reaction medium containing chloroaurate ions, Chandran *et al.* were able to influence the ratio of gold triangular plates to spherical nanoparticles [200]. The study also found that the carbonyl compounds present in the extract assisted in shaping particle growth. While changing the extract concentration modulated particle size between 50 and 350 nm. Furthermore, decahedral, hexagonal, triangular, and spherical Ag nanoparticle shapes have been produced by varying the concentration of *Plectranthus amboinicus* leaf extract in the reaction medium [217].

#### 5.1.3. Influence of Reaction Time

A recent study by Ahmad *et al.* revealed that the reaction time to synthesize spherical Ag nanoparticles using *Ananas comosus* (Pineapple) extract is an important factor indeed. In this particular case it produced a rapid colour change within 2 min [218]. Aqueous Ag(NO)_3_ in the reaction medium was rapidly reduced and nanoparticles appeared within 2 min. The reaction continued up to 5 min, but after that only a slight variation in colour could be observed. The nanoparticles produced were spherical and had a mean size of 12 nm. In a similar study by Dwivedi and Gopal, *Chenopodium album* leaf extract was used to produce Ag and Au nanoparticles. During synthesize nanoparticles appeared within 15 min and continued to form over a 2-h period. Beyond the 2-h period very few nanoparticles were produced [199]. Moreover, a study by Prathna *et al.* revealed that when *Azadirachta indica* leaf extract and Ag(NO)_3_ were combined, increasing the reaction time tended to produce particles with increasing size. The reaction time was varied between 30 min and 4 h to produce a change in particle size ranging from 10 to 35 nm [219].

#### 5.1.4. Influence of Reaction Temperature

While it is generally known that reaction temperature is a crucial factor in any synthesis it has been found that temperature is also an important factor in determining the size, shape, and yield of nanoparticles synthesized via plant extracts [211,220]. For example, synthesis of Ag nanoparticles at a reaction temperature of 25 °C via *Citrus sinensis* (sweet orange) peel extract produced particles with an average size of around 35 nm. However, when the reaction temperature was increased to 60 °C the average particle size decreased to 10 nm [221]. Likewise, Song *et al.* using *Diospyros kaki* (persimmon) leaf extract was able to synthesize stable Ag nanoparticles over a reaction temperature range from 25 to 95 °C. It was also found by Armendariz *et al.*, that thermal variation in the reaction conditions for *Avena sativa* (oat) biomass resulted in changes in the size and shape of Au nanoparticles formed [213]. Additionally, Gericke and Pinches have shown that higher temperatures promote the higher formation rate for Au nanoparticles. At lower temperatures spherical-shaped Au nanoparticles were predominantly formed while at higher temperatures rod-like and plate-like nanoparticles were formed [64]. Reaction rate and particle formation rate appears to become faster when reaction temperature increases, however, the average particle size decreases and particle conversion rate steadily increases with increasing temperature.

### 5.2. Major Nanoparticles Synthesized by Plant Extracts

#### 5.2.1. Gold and Silver Nanoparticles

**Figure 2:**
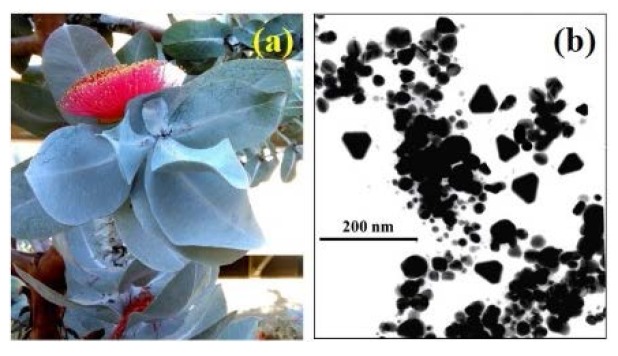
Au nanoparticles synthesised using *Eucalyptus macrocarpa* leaf extract. (**a**) Plant and (**b**) typical transmission electron microscopy image [84].

Au nanoparticles have attracted significant interest due to their size, shape, and surface properties [13,222]. Because of these unique properties, Au nanoparticles have been investigated for potential applications in fields such as biosensors [223,224], hyperthermia therapy [225], delivery platforms for therapeutic drugs and genetic substances [226], and as antibacterial drugs [227,228]. Employing plants as biological factories has the potential to deliver an environmentally friendly source of Au nanoparticles via green chemistry based techniques. For example, Das *et al.* have been able to synthesize spherical shaped Au nanoparticles (~20 nm) using *Nyctanthes arbortristis* (night jasmine) flower extract [229]. While Narayanan and Sakthivel were able to use *Coriandrum sativum* (coriander) leaf extracts to produce Au nanoparticles ranging in size from 7 to 58 nm. The synthesised particles also had diverse shapes such as decahedral, spherical, and triangular [119]. Moreover, several studies have independently reported the synthesis of Au nanoparticles using a variety of plants sources such as the leaves and bark of *Ficus carica* (fig) [230], *Sphaeranthus amaranthoides* [231], and *Putranjiva roxburghii* [232]. Likewise, studies by Armendariz *et al.* have revealed that *Avena sativa* biomass produced Au nanoparticles ranging in size from 5 to 85 nm depending on reaction medium pH. The study also revealed a variety of shapes such as decahedral, hexagonal, isosahedral, irregular, and rod-shaped could be produced depending on reaction medium pH [213]. Also, in a recent study by Poinern *et al. Eucalyptus macrocarpa* leaf extract could be utilised to synthesize Au nanoparticles. The results of this study revealed that spherical particles ranging in size from 20 to 80 nm were the main product. However, coexisting with the spheres were a variety of shapes such as hexagonal pentagon and truncated triangles all ranging in size from 50 to 100 nm as seen in Figure 2 [84].

**Figure 3:**
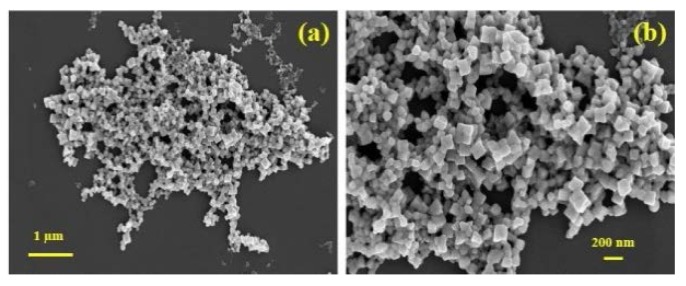
Ag nanoparticles synthesised using *Eucalyptus macrocarpa* leaf extract. (**a**) Overview of agglomerated Ag nanoparticles and (**b**) enlarged view of Ag nanocubes [92].

Historically, Ag is well known for its antimicrobial activity and as a result it is commonly used in a variety of medical preparations against pathogens [233,234,235]. For antimicrobial preparations, the size and high surface area to volume ratio of Ag nanoparticles enables them to closely interact with the bacterial cell membranes [236]. Recent antimicrobial studies have revealed that significant membrane damage and DNA toxicity can result from the interaction between Ag nanoparticles via bio-sorption and cellular uptake [31,237]. Among biological synthesis processes, plants are found to be more conducive and provide a faster pathway for manufacturing Ag nanoparticles compared to conventional microbial processes. For example, Edison and Sethuraman have used *Terminalia chebula* (harad) fruit extract to rapidly produce Ag nanoparticles [238]. Likewise, Poinern *et al.* have also used *Eucalyptus macrocarpa* leaf extract to synthesize cubic Ag nanoparticles ranging in size from 50 to 200 nm as seen in Figure 3 [92]. Studies by Geetha *et al.* have shown high antibacterial efficacy of Ag nanoparticles synthesized using *Cymbopogan citratus* (lemon grass) leaf extract. The Ag nanoparticles ranged in size from 15 to 65 nm with an average size of 34 nm. The shape of the particles was predominately cuboidal and rectangular. The antibacterial effect was found to be effective against *Pseudomonas aeruginosa*, *Proteus mirabilis*, *Escherichia coli*, *Shigella flexaneri*, *Shigella sonnei,* and *Klebsiella pneumonia* [239,240].

In addition to pure metal nanoparticles being synthesized by plants, several authors have also reported alloying Au and Ag to investigate the properties of the resulting bimetallic nanoparticle. Bimetallic nanoparticle synthesis involves the competitive reduction between two aqueous solutions each containing a different metallic ion precursor that is mixed with a plant extract. In the case of an Au-Ag bimetallic nanoparticle, Au having the larger reduction potential will form first to create the core of a resulting core-shell structure. Subsequent reduction of Ag ions results in Ag coalescing on the core to form the shell. Plants that have been successfully used to synthesize Au-Ag bimetallic nanoparticles include *Azadirachta indica* (neem) [79], *Anacardium occidentale* (cashew nut) [241], *Swietenia mahagony* (West Indies mahogany) [242], and cruciferous vegetable extracts [243].

#### 5.2.2. Copper and Copper Oxide Nanoparticles

Copper (Cu) and copper oxide (CuO) nanoparticles have been synthesized by a variety of plant extracts. Cu nanoparticles have been biologically synthesized using magnolia leaf extract to produce stable nanoparticles ranging in size from 40 to 100 nm. Antimicrobial studies revealed that the Cu nanoparticles have potential antibacterial activity against *Escherichia coli* cells, a common pathogen [30]. *Syzygium aromaticum* (Clove) extracts can produce Cu nanoparticles with a mean particle size of 40 nm and a spherical to granular morphology [244]. Cu nanoparticles can be synthesised using stem latex of *Euphorbia nivulia* (Common milk hedge). These nanoparticles are coated and stabilized by peptides and terpenoids present in the latex; these nanoparticles are reported to be toxic to human adenocarcinomic alveolar basal epithelial cells (A549 cells) [245,246]. Furthermore, a study by Padil *et al.* using *Sterculia urens* (Karaya gum) extract was able to synthesize highly stable spherical Cuprous Oxide (CuO) nanoparticles with a mean particle size of 4.8 nm. The particles were found to have significant antimicrobial activity against common pathogens such as *Escherichia coli* and *Staphylococcus aureus* [247]. Similar studies have also shown that CuO nanoparticles exhibit both antioxidant and antibacterial behaviour [248,249].

#### 5.2.3. Palladium and Platinium Nanoparticles

Palladium nanoparticles were synthesised by Satishkumar *et al.* in 2009, using an extract of taken from *C. zeylanicum* (cinnamon) bark [215]. Changing the bark extract concentration, reaction pH and temperature during synthesis was found not to influence particle size (15 to 20 nm) and morphology. Palladium nanoparticles ranging in size from 75 to 85 nm have also been synthesized using *Annona squamosa* (Custard apple) peel extract [250], while the leaf extract of soybean (*Glycine max*) have been able to synthesise nanoparticles with a mean size of 15 nm [251]. And even common commercial products like *Coffea arabica* (Coffee) and *Camellia sinensis* (Tea) extracts have been utilised to synthesise palladium nanoparticles varying in size from 20 to 60 nm with faced centred cubic crystal symmetry [252]. Moreover, when an extract taken from *Gardenia* jasminoides (Cape jasmine)was used to synthesise palladium nanoparticles, antioxidants such as geniposide, chlorogenic acid, crocins, and crocetin were found to act as both reducing and stabilizing agents [253]. Subsequent analysis revealed particle sizes ranged from 3 to 5 nm and the study also found particle size was dependent on reaction temperature.

The first synthesis of platinium nanoparticles was reported by Song *et al.*, in 2010 using a leaf extract taken from *Diospyros kaki* (Persimmon). The resultant nanoparticles ranged in size from 2 to 12 nm and showed that 90% of the platinum ions in solution were converted using a 10% concentration of leaf biomass at 95 °C [204]. A leaf extract taken from *Ocimum sanctum* (Holy basil) has also been used to synthesise platinum nanoparticles with a mean particle size of 23 nm from aqueous chloroplatinic acid at a reaction temperature of 100 °C [254]. And recently, the biological synthesis of platinum nanoparticles with particle size and shape control has also been reported by using plant wood nanometre scale materials [255]. For example, Coccia *et al.* have recently reported a one-pot synthesis technique for producing platinum and palladium nanoparticles using lignin isolated from red pine (*Pinus resinosa*) [25].

#### 5.2.4. Titanium Dioxide and Zinc Oxide Nanoparticles

A number of plant extracts have been also been found to synthesize important metal oxide nanomaterials such as titanium dioxide (TiO_2_) and zinc oxide (ZnO) nanoparticles. For example, Roopan *et al.* have found that *Annona squamosa* peel could be used to effectively synthesize TiO_2_ nanoparticles [256], while *Nyctanthes arbor-tristis* leaf extracts have been found to produce spherical particles ranging in size from 100 to 150 nm [257] and *Eclipta prostrata* leaf extracts can produce particles ranging in size from 36 to 68 nm [258,259]. Velayutham *et al.* have used a *Catharanthus roseus* leaf extract to biologically synthesize TiO_2_ nanoparticles. The resultant nanoparticles were irregular in shape and ranged in size from 25 up to 110 nm. Assessment of the resulting TiO_2_ suspensions revealed that they were both adulticidal and larvicidal against *Hippobosca maculate* (hematophagous fly) and *Bovicola ovis* (sheep louse) [260]. The antibacterial and antioxidant properties of TiO_2_ nanoparticles synthesized via an extract from *Psidium guajava* were evaluated against *Aeromonas hydrophila*, *Proteus mirabilis*, *Escherichia coli*, *Staphylococcus aureus,* and *Pseudomonas aeruginosa* pathogens [261]. The nanoparticles were found to be most effective against *Staphylococcusaureus and Escherichia coli*. Furthermore, the antibacterial and antioxidant properties of nanometre scale and bulk TiO_2_ towards bacteria have also been examined and found to be deleterious towards a number of bacterial strains [249].

Zinc oxide nanoformulations is an important biomedical and cosmetic product. The latex from *Calotropis procera* has been used as both reducing and stabilizing agent for the synthesis of spherical shaped zinc oxide (ZnO) nanoparticles [262]. While stable and spherical ZnO nanoparticles have been synthesized using *Aloe vera* extract [263]. In addition, crystalline poly-dispersed ZnO nanoparticles with a mean particle size of 72.5 nm were synthesized via *Physalis alkekengi* extract [264] and nanoparticles synthesized from Sedum alfredii were pseudo-spherical in shape with a mean particle size of 53.7 nm. [265]. A recent study by Vimala *et al.* has shown the ability of green synthesized ZnO nanoparticles to be used as drug delivery platforms for doxorubicin, which highlights the importance of developing novel green chemistry based techniques for developing new sources of nanoparticles [266].

#### 5.2.5. Indium Oxide, Iron Oxide, Lead, and Selenium Nanoparticles

A number of other types of metal and metal oxide nanoparticles have been biologically synthesized using a variety of plants. Leaf extracts from *Aloe vera* (*Aloe barbadensis* Miller) have been used to synthesize Indium oxide (In_2_O_3_) nanoparticles. After initial synthesis, the precipitate was thermally treated between 400 and 600 °C to produce the nanoparticles. The resultant spherical nanoparticle size was dependent on treatment temperature and ranged from 5 to 50 nm [201]. Because of the importance of Iron (Fe) nanoparticles in a number of environmental remediation technologies, recent research has focused on green chemistry based methods to synthesize these Fe nanoparticles. For example, aqueous sorghum bran extracts have been used to biologically synthesize Fe nanoparticles at room temperature [29]. Recently Pattanayak *et al.* were able to synthesize spherical Fe nanoparticles with a mean particle size of 100 nm via leaf extracts taken from *Azadirachta indica* (Neem) [267]. And a short time ago Shah *et al.* were able to synthesise Fe nanoparticle via extracts taken from plants such as *Euphorbia milii*, *Tridax procumbens*, *Tinospora cordifolia*, *Datura innoxia*, *Calotropis procera,* and *Cymbopogon citratus* (lemon grass tea). The smallest spherical nanoparticles size range (13 to 21 nm) were synthesized from the stem extract taken from *Euphorbia milii* and the widest size range (43–342 nm) occurred for particles synthesized using leaf extracts taken from *Cymbopogon citratus* [268]. Other significant metallic nanoparticles that have been biologically synthesized include lead (Pb) and selenium (Se). In the case of Pb nanoparticles, Joglekar *et al.* were able to use the latex from *Jatropha curcas* to synthesise spherical shaped particles ranging in size from 10 to 12.5 nm [269]. Recently, Sasidharan *et al.* were able to synthesise Selenium (Se) nanoparticles using the extracts taken from the peel of *citrus reticulata* to produce spherically shaped particles with a mean particle size of 70 nm [270].

## 6. Applications of Nanoparticles & Biologically Inspired Templates

The continually developing field of nanotechnology is expected to require a significant amount of optimised and functional nanomaterials. A wide range of conventional physicochemical processes has been used in the recent past to synthesise a wide variety of metal nanoparticles. These nanoparticles have been used in a diverse range of applications such as biosensors [271], targeted drug delivery platforms [10,14,272], diagnostics and therapeutics [273], cancer treatments [9,274], pesticides [275], and antimicrobials [276]. However, nanoparticles produced by environment-friendly biological entities have only been exploited in relatively few practical applications. Ag nanoparticles have attracted considerable research interest due to its inherent antimicrobial activity and as a result it is already used as an antimicrobial agent in a wide range of commercially available medical and consumer products [18,20,277]. Another emerging application of nanoparticles and Ag nanoparticles in particular is in crop protection and the management of agricultural plant diseases [278,279]. Recent studies by Vivek *et al.* have demonstrated the antifungal effects of Ag nanoparticles [280]. Futhermore, Ag nanoparticles can be used to control a number of plant pathogens in a safer way compared to conventional fungicides [281] and these metallic Ag nanoparticles have also been found to be active against cancer cells and plasmodial pathogens [282,283,284,285].

Traditionally, Au has been used in several medical applications. Au nanoparticles have attracted significant interest over the last decade as a medicinal material in treatment of tumours. For example, Au nanoparticles have the ability to passively accumulate in tumours due to their size and because of their unique optical and chemical properties can be used in thermal treatment procedures [286,287]. Moreover, studies have shown that biocompatible Au nanoparticles can be successfully used as carrier platforms for the targeted delivery of anticancer drugs thus improving delivery and minimizing treatment durations and side effects [13,226,288]. Studies have also shown that Au nanoparticles are effective antibacterial agents against a number of bacterial strains [84,289]. While Cu and CuO nanoparticles have also been found to be strong antimicrobial agents and their disinfecting properties against a number of infectious organisms means they can be used as an effective bactericide material to coat hospital equipment [244,290,291,292]. Pt nanoparticles have the potential to be used in water electrolysis applications [254]. TiO_2_ nanoparticles, because of their antibacterial activity, have been used in antibacterial coatings and wastewater disinfection processes [293,294,295]. While ZnO nanoparticles display good antibacterial activity and have been used in food packaging and wastewater treatments [296,297]. Moreover, template assisted fabrication using biological entities permits the creation of more complex self-assembled structures at both the nanometre and micrometre scales. Bacteria, bacteriophages and viruses are attractive assemblers for manufacturing one dimensional structures into ordered arrays. For example, the tobacco mosaic virus has been used to assemble Au, Ag and Pt nanoparticles [298] and filamentous bacteriophages have been used to form silica fibres and nanotubes [299,300,301,302]. These nanometre scale entities are very effective templates for forming well-ordered 1D assemblies [303,304]. While entities such as silk sericin have been used to form nano-fibrous networks that direct the formation of needle like hydroxyapatite particles [305] and promote osteogenic properties of human bone marrow cells [306]. While magnetically controlled guidance of biomolecules via iron oxide nanoparticles has been able to produce high ordered 3D arrays used to support stem cell growth [307]. Furthermore, films incorporating Au nanoparticles have been assembled from genetically engineered bacteria and filamentous viruses to produce CdS quantum dots [308] and colourimetric sensors [309,310]. Recent studies by Wang *et al.* have demonstrated that viral nanofibres decorated with magnetic iron oxide nanoparticles can be used for the detection of human serum antibody biomarkers [311]. Nanoparticles and nanoparticle constructed structures have the potential to be used in a wide variety of applications as discussed above, especially if they can be synthesised using biological entities that can ensure clean, nontoxic, and eco-friendly methods of production.

The synthesis of metallic nanoparticles using a wide variety of biological entities, as discussed above, has been actively pursued in recent years as an alternative bottom up approach to self-assemble atoms to form nuclei and subsequently grow into nanometre scale particles. However, several factors have been identified that can significantly influence the viability of this eco-friendly process for synthesising nanoparticles. The most readily identified factors being particle size control, shape, and size distribution. These factors are all directly influenced by reaction medium pH, reactant moieties, reactant concentrations, reaction time, and temperature. As explained above, even small variations in these factors can significantly influence particle size, shape, and size distribution. For example, in the case of plant extracts, there can be noticeable variations in the chemical composition of extracts taken at different times of the year and at different locations around the world for the same species. This compositional variation can often lead to different laboratories producing dissimilar results from the same plant extract and metal salt. This can be a serious drawback in using plant extracts to produce nanoparticles with consistent physical and chemical properties. Understandably, even with the current limitations, biosynthesis offers numerous advantages and has the potential to deliver nanoparticles with predetermined properties. For example, Shankar *et al.*, using effective quality control and closely regulating the reaction medium pH, reactant concentrations, reaction time, and temperature during synthesis were able to reduce large quantities of triangular Au nanoprisms using *Cymbopogon flexuosus* (Lemongrass) extract [312]. Nearly 45% of the total nanoparticles reduced from the aqueous chloroaurate ion and extract solution was composed of Au nanotriangles. The triangles displayed truncated vertices similar to those seen for triangular Ag [313] and Au nanoprisms [314] synthesised by chemical and photochemical methods. Furthermore, repeated centrifugation (3400 g), washing, and re-dispersion of the reaction medium significantly improved the throughput of nanotriangle numbers (up to 90%). Interestingly, despite recent developments in conventional physical and chemical methods, many physical methods still require relatively expensive equipment and have operational requirements such as vacuum, pressurized gases, and high temperatures. While most chemical methods tend to use toxic materials such as organic solvents, reducing agents, and stabilizers. These economic and toxicity related emphasizes the importance and need for further research into eco-friendly biosynthesis methods factors further over the more traditional nanoparticle production processes.

## 7. Conclusions

Nanoparticles, in particular metallic nanoparticles have attracted considerable interest in many and diverse fields such as electronics, photonics, medicine, and agriculture. This review has summarized recent research into the synthesis of metallic nanoparticles using biological entities. However, owing to the diversity of biological entities ranging from microorganisms to plants, much of this field remains largely unknown and still remains to be discovered. The production of nanoparticles using biological entities has the potential to deliver new sources of novel materials that are stable, nontoxic, cost effective, environment-friendly, and synthesized using green chemistry approach. This green chemistry approach of using biological entities is in complete contrast with conventional chemical and physical processes that often use toxic materials that have the potential to cause environmental toxicity, cytotoxicity, and carcinogenicity. Whilst biological entities have been extensively used to produce nanoparticles, the use of plants offers a straightforward, clean, non-toxic, and robust procedure that does not need any special culture preparation or isolation techniques that are normally required for bacteria and fungi based techniques. In particular, the use of plant extracts for synthesizing nanoparticles is inexpensive, easily scaled up, and environment-friendly. Plant extracts have the potential to produce nanoparticles with a specific size, shape and composition. Plant synthesized nanoparticles have the potential to be widely used in current medical procedures involving nanoparticles such as fluorescent labelling in immunoassays, targeted delivery of therapeutic drugs, tumour destruction via heating (hyperthermia), and as antibacterial agents in bandages. On another front, plant synthesized nanoparticles have the potential to be used for the delivery of anti-microbiological compounds for use as pesticides for agricultural crops. Moreover, agricultural crop wastes and food industry wastes are also excellent candidates for supplying sources of plant-based bio-chemicals with the potential to synthesize metallic nanoparticles and similar products. Despite the environmental advantages of using green chemistry based biological synthesis over traditional methods as discussed in this article there are some unresolved issues such as particle size and shape consistency, reproducibility of the synthesis process, and understanding of the mechanisms involved in producing metallic nanoparticles via biological entities. In the case of plant extracts, nanoparticle formation mechanisms vary between different plant species. Therefore, there is a need for more studies to evaluate and understand the actual plant dependent mechanisms. This is a grossly unexplored field and requires much more research investment to fully utilize the green synthesis of metallic nanoparticles via biological entities.
